# Use of machine learning-based integration to develop a monocyte differentiation-related signature for improving prognosis in patients with sepsis

**DOI:** 10.1186/s10020-023-00634-5

**Published:** 2023-03-20

**Authors:** Jingyuan Ning, Keran Sun, Xuan Wang, Xiaoqing Fan, Keqi Jia, Jinlei Cui, Cuiqing Ma

**Affiliations:** 1 Department of Immunology, Hebei Medical University, Shijiazhuang, People’s Republic of China; 2 Department of Laboratory, The Second Hospital of Hebei Medical University, Shijiazhuang, People’s Republic of China; 3 Department of Pathology, Shijiazhuang People’s Hospital, Shijiazhuang, People’s Republic of China

**Keywords:** Sepsis, Single cell, Machine learning, Prognosis, EVL

## Abstract

**Background:**

Although significant advances have been made in intensive care medicine and antibacterial treatment, sepsis is still a common disease with high mortality. The condition of sepsis patients changes rapidly, and each hour of delay in the administration of appropriate antibiotic treatment can lead to a 4–7% increase in fatality. Therefore, early diagnosis and intervention may help improve the prognosis of patients with sepsis.

**Methods:**

We obtained single-cell sequencing data from 12 patients. This included 14,622 cells from four patients with bacterial infectious sepsis and eight patients with sepsis admitted to the ICU for other various reasons. Monocyte differentiation trajectories were analyzed using the “monocle” software, and differentiation-related genes were identified. Based on the expression of differentiation-related genes, 99 machine-learning combinations of prognostic signatures were obtained, and risk scores were calculated for all patients. The “scissor” software was used to associate high-risk and low-risk patients with individual cells. The “cellchat” software was used to demonstrate the regulatory relationships between high-risk and low-risk cells in a cellular communication network. The diagnostic value and prognostic predictive value of Enah/Vasp-like (*EVL*) were determined. Clinical validation of the results was performed with 40 samples. The “CBNplot” software based on Bayesian network inference was used to construct *EVL* regulatory networks.

**Results:**

We systematically analyzed three cell states during monocyte differentiation. The differential analysis identified 166 monocyte differentiation-related genes. Among the 99 machine-learning combinations of prognostic signatures constructed, the Lasso + CoxBoost signature with 17 genes showed the best prognostic prediction performance. The highest percentage of high-risk cells was found in state one. Cell communication analysis demonstrated regulatory networks between high-risk and low-risk cell subpopulations and other immune cells. We then determined the diagnostic and prognostic value of *EVL* stabilization in multiple external datasets. Experiments with clinical samples demonstrated the accuracy of this analysis. Finally, Bayesian network inference revealed potential network mechanisms of *EVL* regulation.

**Conclusions:**

Monocyte differentiation-related prognostic signatures based on the Lasso + CoxBoost combination were able to accurately predict the prognostic status of patients with sepsis. In addition, low *EVL* expression was associated with poor prognosis in sepsis.

**Supplementary Information:**

The online version contains supplementary material available at 10.1186/s10020-023-00634-5.

## Introduction

Sepsis is a fatal organ dysfunction attributed to host response disorder to severe infection. It is one of the major causes of death in critical patients. The condition of patients with sepsis changes rapidly. Therefore, early diagnosis and intervention may help improve the prognosis of patients with sepsis (Arina and Singer 2021). At present, many studies are examining various biomarkers measured by a variety of different technologies to quickly distinguish systemic inflammatory response syndrome (SIRS), which is an over-defensive source of harmful body pressure (such as infection, trauma, surgery, acute inflammation, blood deficiency/reperfusion, or cancer) (Chakraborty and Burns 2022) or early identification of organ dysfunction (sepsis) caused by infection. Biomarkers may help to stratify patients with sepsis based on biological phenotypes, such as high inflammation and immunosuppression. Biomarkers can also be used to evaluate intestinal permeability, blood–brain barrier (BBB) permeability, readmission probability, and long-term outcomes (Yende et al. 2019; Barichello et al. 2021).

The main cause of sepsis is the replication and release of components by pathogenic pathogens, such as endotoxin and exotoxin, and DNA. These components are called pathogen-associated molecular patterns (PAMPs) (Kumar et al. 2013; Heckenberg et al. 2014). PAMPs are recognized by pattern recognition receptors (PRR) as well as non-PRRs (Mook-Kanamori et al. 2011; Sellner et al. 2010). The recognition of PAMPs by a variety of immune cell receptors triggers a series of signal pathways, which activate a variety of transcription factors to promote the production and release of proinflammatory and anti-inflammatory mediators, which are necessary to eliminate invading pathogens (Iwasaki and Medzhitov 2010).

Both host immune response and pathogen virulence factors can cause cell damage and/or induce cell stress. Many damage-associated molecular patterns (DAMPs) have been identified, some of which are currently used as biomarkers of inflammation. Examples include heat shock protein (HSP), high mobility group box 1 (HMGB-1), and members of the S100 family (Kataoka et al. 2014; Kigerl et al. 2014; Wiersinga et al. 2014). The immune response may induce vascular endothelial damage and may promote the transfer of the pathogen and/or its PAMP from the intestinal tract to the bloodstream and lymphatic vessels, thereby amplifying the systemic inflammatory response (Generoso et al. 1999).

**Fig. 1:**
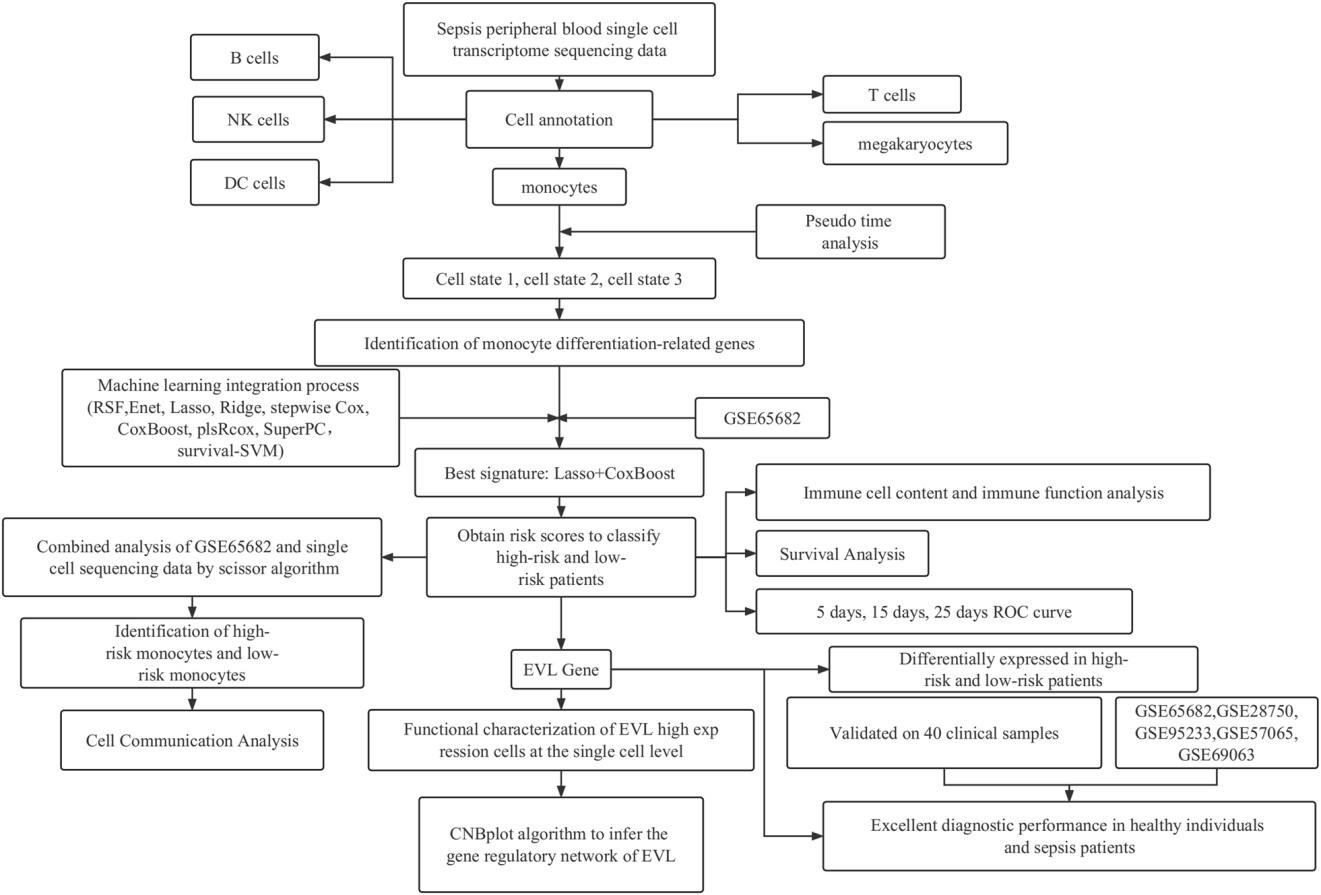
All analytical processes in this study

Past studies have explored biomarkers that can help identify endothelial damage, intestinal permeability, organ failure, and BBB decomposition and predict readmission, short-term and long-term mortality, and the cognitive consequences in survivors (Barichello et al. 2019). However, molecular mechanisms underlying sepsis have not been fully elucidated. To identify suitable biomarkers for the early detection and treatment of sepsis, we collected sequencing data of peripheral blood single cells derived from patients with sepsis. The differentiation trajectories of monocytes was analyzed, and the differentiation-related genes were identified. Based on the expression of differentiation-related genes, 99 machine-learning combinations of prognostic signatures were derived, and the best machine-learning algorithm was used to calculate the risk scores of all patients. The validity and accuracy of the best signature were systematically analyzed. In addition, Enah/Vasp-like (*EVL*) has been implicated in cancer, cardiovascular disease, and neurological disorders. *EVL* is involved in regulating processes such as cytoskeletal dynamics, cell division, cell migration, and intercellular communication. However, the diagnostic and prognostic value of *EVL* in sepsis remains unclear. In our study, we validated the strong diagnostic performance of *EVL* in differentiating sepsis from healthy individuals across multiple datasets and 40 clinical samples. Furthermore, we observed significant differential expression of *EVL* between high- and low-risk patients. Overall, our study provides insights for clinical diagnosis and treatment.The flow of all the analyses in this study is shown in Fig. 1

## Materials and methods

### Acquisition and pre-processing of single-cell data

Single-cell transcriptome data were obtained from the Single Cell Portal (https://singlecell.broadinstitute.org/single_cell/study/SCP548/an-immune-cell-signature-of-bacterial-sepsis-patient-pbmcs#study-summary). Quality control was performed in R(4.1.2) environment using standard single cell processing procedures. The count matrix were read using the Read10X function from the Seurat package (Version 4.0.4), and the latter was further converted to dgCMatrix format. The merge function was used to integrate all individual objects into an aggregate object, and the RenameCells function was used to ensure that all cell labels were unique. We filtered low quality cells with the following filtering criteria: when a gene was expressed in less than 3 cells, the gene was deleted. When the number of genes expressed in a cell was less than 200, the cell was deleted. A global-scaling normalization method (“LogNormalize”) was employed to ensure that the total gene expression in each cell was equal, and the scale factor was set to 10,000. The top 2000 variably expressed genes were returned for downstream analysis using the FindVariableFeatures function. The ScaleData function, “vars.to.regress” option UMI, and percent mitochondrial content were used to regress out unwanted sources of variation. Principal component analysis (PCA) incorporating highly variable features reduced the dimensionality of this dataset, and the first 30 PCs were identified for analysis. Harmony method (Korsunsky et al. 2019) was used to remove batch effects between samples. Cells were down-dimensioned using the UMAP method. Clustering analysis was performed based on the edge weights between any two cells, and a shared nearest-neighbor graph was produced using the Louvain algorithm, which was implanted in the FindNeighbors and FindClusters functions. The parameter of resolution in the FindClusters function was tried repeatedly between 0.1 and 1. Cell clustering trees at different resolutions were observed using the clustree function, and the results showed that the clearest clustering results were obtained when the resolution was 0.5. To annotate the cell clusters, differentially expressed markers of the resulting clusters were identified with the FindAllMarkers function using the default nonparametric Wilcoxon rank sum test with Bonferroni correction. All cells were annotated according to cell surface markers and annotated genes used in the relevant literature and CellMarker database (Zhang et al. 2019) (http://xteam.xbio.top/CellMarker/).

### Pseudo time analysis

The “monocle” package (2.24.1) was used to perform the proposed pseudo time analysis (Trapnell et al. 2014).The NewCellDataSet function was used to create a new object for the monocle using transcript count data. Signature genes expressed in at least 10% cells of the dataset and with a P < 0.01 calculated using the differentialGeneTest function were included to define the trajectory progress. The ReduceDimension function reduced the space down to two dimensions, and the orderCells function ordered the cells according to gene expression. After the orderCells function is run, the monocyte states 1, 2 and 3 are available. The FindAllMarkers function in the “seurat” package was used to determine the genes characteristic of each differentiation state. If |log2FC| > 0.585, an adjusted P value < 0.05 was considered as a differentially expressed gene.

### Machine learning to build prognostic signatures

We used a total of 10 machine learning algorithms, including random survival forest (RSF), elastic network (Enet), Lasso, Ridge, stepwise Cox, CoxBoost, partial least squares regression for Cox (plsRcox), supervised principal components (SuperPC), generalised boosted regression (GBM), and survival support vector machine (survival-SVM). In the process, we use one algorithm to filter the variables and another algorithm to build the prognostic signature. Out of the possible 100 combinations of machine learning algorithm pairs, one was excluded because the number of genes included in the final prognostic signature was less than five. A total of 99 combinations of machine learning algorithms were eventually integrated. Finally, the Harrell’s concordance index (C-index) was calculated for each signature, and the signature with the highest average C-index value was considered to be the best signature. After calculating the risk score for each patient using the predict function, the optimal cutoff value for the risk score is determined using the surv_cutpoint function in the “srvminer” package. Based on the optimal cutoff value of the risk score, patients are divided into high-risk and low-risk groups.

### Identification of phenotypic-related cells

Scissor algorithm from the “Scissor” package (Sun et al. 2022) (2.0.0). By leveraging bulk data and phenotype information, this algorithm automatically selects cell subpopulations from single-cell data that are most responsible for the differences of phenotypes. The novelty of Scissor is that it utilizes phenotype information from bulk data to identify the most highly disease-relevant cell subsets. In our study, high-risk patients and low-risk patients identified in GSE65682 were treated as two different phenotypes. Based on the scissor algorithm, the high-risk phenotype and the low-risk phenotype were associated with each monocyte. Parameter α balances the effect of the L1-norm and the network-based penalties. A larger alpha inclines to emphasize the L1-norm to encourage sparsity, making Scissor selects fewer cells than the result using a smaller alpha. According to the requirements of the scissor algorithm: the number of Scissor selected cells should not exceed a certain percentage of total cells (default 20%) in the single-cell data, we finally choose an alpha value of 0.5. To determine whether the inferred phenotype-to-cell associations are reliable, we use the Reliability.Test function to perform a reliability significance test. The motivation for the reliability significance test is: if the chosen single-cell and bulk data are not suitable for the phenotype-to-cell associations, the correlations would be less informative and not well associated with the phenotype labels. Thus, the corresponding prediction performance would be poor and not be significantly distinguishable from the randomly permutated labels. If the P-value of the test was found to be less than 0.05, the inferred phenotype-cell association was reliable.

### Cellular communication network

Cell–cell interaction analysis was performed based on the “CellChat” (v1.0.0) R package (Jin et al. 2021). CellChat has a public repository of ligands, receptors, cofactors and their interactions (http://www.cellchat.org/). The CellChat R package is a versatile and easy-to-use toolkit for inferring, analyzing, and visualizing cell–cell communication from any given scRNA-seq data. The ligand and receptor genes expressed by each cell were projected into a manually selected reference communication network and the probability of communication in each pathway was inferred by gene expression. Finally use the netVisual_bubble function for visualization, with all parameters as default.

### Enrichment analysis

Enrichment analysis of differential genes was performed using the “GSEABase” package, “ClusterProfiler” package and “org.Hs.eg.db” package. The database used for the enrichment analysis was derived from the Gene Ontology (http://geneontology.org/). Use the EnrichGO function for enrichment. If P < 0.05, the pathway was considered to be significantly enriched. “ggplot2” package, “ggpubr” package for visualization.

### Inference of gene interaction networks

When investigating gene expression profiles, identifying important directional edges between genes can provide valuable insights in addition to identifying differentially expressed genes. The “CBNplot” package (Sato et al. 2022) for the inference of gene interaction networks was used to calculate the gene interaction networks. Sequence data from 761 sepsis patients in the GSE65682 data were used as background to calculate the interaction direction and plot the graph using the bngeneplot function, with a filtering criterion of 0.95 as the intensity.

### Analysis of immune cell content and immune function

Immune cell content and immune function analysis was performed using the gsva function in the “GSVA” package. By default, kcdf = “Gaussian” which is suitable when input expression values are continuous, such as microarray fluorescent units in logarithmic scale, RNA-seq log-CPMs, log-RPKMs or log-TPMs. Therefore, the parameters method choose ssgsea and kcdf choose Gaussian. Use ggpubr to visualize the results.

### Clinical sample collection

In total, 20 patients with sepsis (mean age, 58 years; 66%men) and 20 age-and sex-matched healthy controls (mean age, 56 years; 66%men) were included. The diagnosis of sepsis was established according to the Third International Consensus Definitions for Sepsis and Sepsis Shock (Sepsis-3). Sepsis was defined as a documented or suspected infection (defined as a pathologic process induced by a microorganism) and some of the following parameters (Table 1). The study was approved by the Institutional Review Boards of The Second Hospital of Hebei Medical University.

**Table 1:** Inclusion criteria for sepsis

General parameters
• Hyperthermia (> 38.3 °C) or hypothermia (< 36.0 °C)
• Tachycardia (heart rate > 90 beats/min)
• Tachypnea (respiratory rate higher than 30 breaths/min)
• Altered mental status
• Significant edema or positive fluid balance > 0 ml/kg over a 24-h period)
• Hyperglycemia in the absence of diabetes (plasma glucose > 110 mg/dl)
Inflammatory parameters
• Lleukocytosis or leukopenia (white blood cell count > 12,000/mm^3^ or < 4000 mm^3^), normal white blood cell count with a percentage of immature forms > 10%
• Plasma C-reactive protein more than 2 standard deviations above the normal value
• Plasma procalcitonina more than 2 standard deviations above the normal value
Hemodynamic parameters
• Arterial hypotension: systolic blood pressure < 90 mm Hg, mean arterial blood pressure < 70 mm Hg, or decrease of systolic blood pressure from the baseline to > 40 mm Hg)
• Mixed venous oxygen saturation > 70%
• Cardiac index > 3.5 l/min/m^2^
Organ dysfunction
• Arterial hypoxemia (pressure of arterial oxygen/fraction inspired oxygen (PaO_2_/FIO_2_) ratio < 300)
• Acute oliguria (urine output < 0.5 ml/kg/h for at least 2 h)
• Creatinine increase of 0.5 mg/dl or more
• Coagulation abnormalities defined as international normalized ratio (INR) > 1.5 or activated partial thromboplastin time (APTT) > 60 s
• Ileus (absent bowel sounds)
• Thrombocytopenia (platelet count < 100,000/μl)
• Hyperbilirubinemia (plasma total bilirubin > 4 mg/dl)
Tissue perfusion parameters
• Hyperlactatemia (> 3 mmol/l)
• Decreased capillary refill or mottling

### Quantitative reverse transcription polymerase chain reaction (qRT-PCR)

PBMCs were isolated from whole blood using a standard Ficoll-Paque isolation method. Total RNA from the PBMC was isolated using TRIzol reagent (Invitrogen, Carlsbad, California, USA), and qRT-PCR was performed with SYBR® Green dye (TaKaRa, Shiga, Japan), following the manufacturer’s instructions. Primer sequences: EVL: F-CAGCAGCAGCGTCAGGAATCTC; R-GTGGGTGGAGGTGGGACTGG. GAPDH: F-AGAACATCATCCCTGCCTCTACT; R-GATGTCATCATATTTGGCAGGTT. GAPDH was used as a reference gene.

### Statistical analysis

All statistical analyses were carried out using R (4.1.2) and Strawberry Perl software (5.14.2.1, 64-bit).

## Results

### Analysis of monocyte differentiation trajectories

**Fig. 2:**
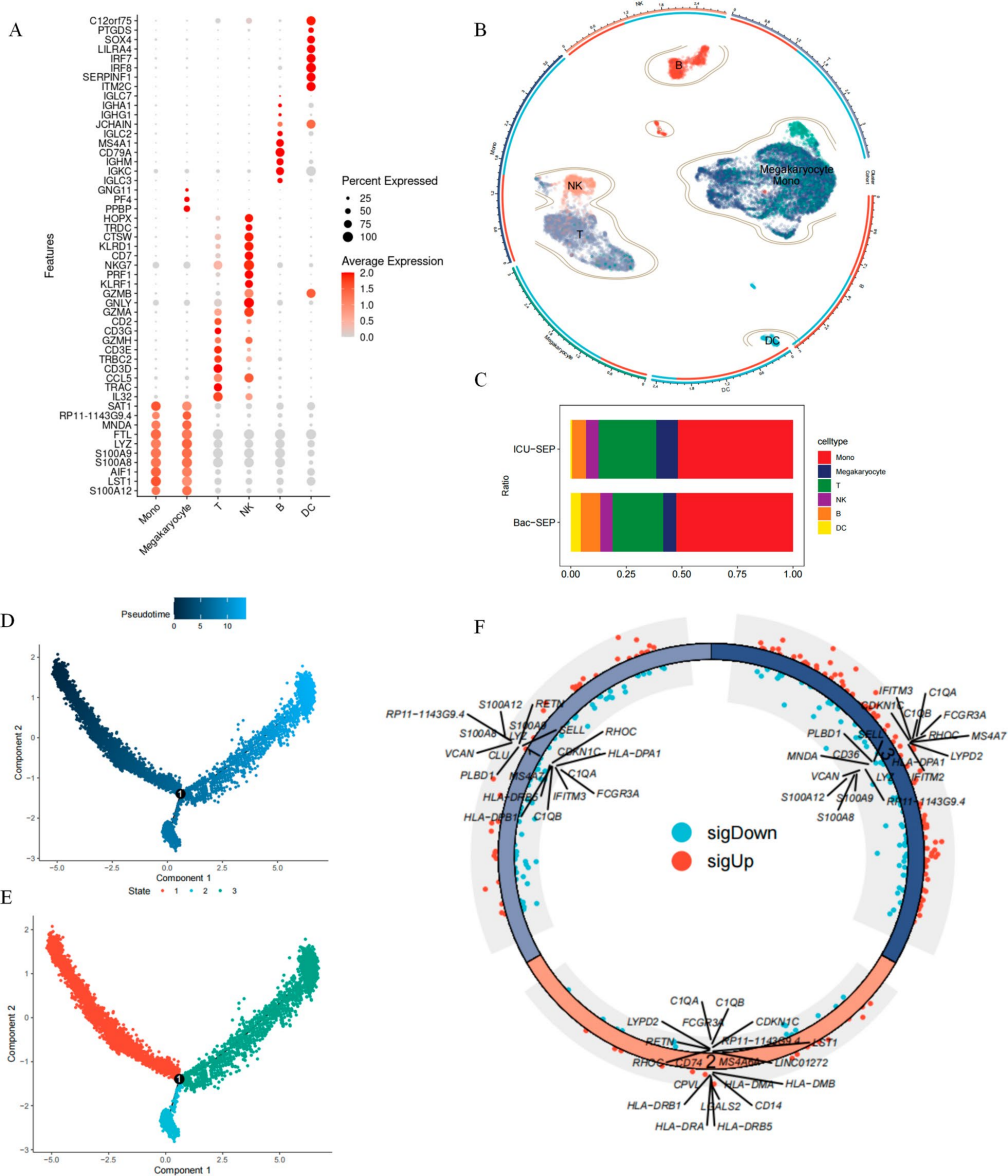
Analysis of monocyte differentiation trajectories. **A** Expression of marker genes used in the cell annotation process. The size of the bubble represents the percentage of cells in that cell subpopulation that express the gene. From gray to red indicates higher gene expression values; **B** Landscape of single-cell annotation; **C** Percentage of each cell type in different cohorts; **D** Monocyte differentiation trajectories. The pseudotime represents the order of differentiation time, the darker the color means the more biased to the early stage of differentiation, the lighter the color, the more backward the differentiation time sequence; **E** Monocyte differentiation states. Red represents state 1 cells, blue represents state 2 cells, and green represents state 3 cells. Cells start to differentiate from state 1, after which they partly differentiate to state 2 and partly to state 3; **F** Differentially expressed genes for each cell state. Differentially expressed genes were determined for each state by a Wilcoxon rank-sum test, with a screening bar of |logFC| > 0.585 adjusted for P < 0.05. Blue represents down-regulated genes, red represents up-regulated genes

We first obtained peripheral blood single-cell sequencing data for a total of 14,622 cells obtained from four patients with bacterial infection-related sepsis and eight patients with sepsis admitted to the ICU for various reasons. We annotated all cells according to common cell surface markers (Fig. 2A). The annotation results showed that six different cell types, including B cells, NK cells, monocytes, DC cells, T cells, and megakaryocytes (Fig. 2B). Cell proportion analysis showed that monocytes accounted for more than 50% of both cohorts (Fig. 2C). Since monocytes were the largest cell subpopulation, we extracted only monocytes for subsequent analysis. We performed a pseudo time analysis of 7631 monocytes (Fig. 2D). The results showed three different differentiation states of monocytes among patients with sepsis (Fig. 2E). We determined the differentially expressed genes in each cell state compared to other cell states, and a total of 166 differential genes were included using |log2FC| > 0.585 adjusted for P < 0.05 as a screening condition (Fig. 2F). These genes were termed monocyte differentiation-related genes found in patients with sepsis. We found that the expression of pro-inflammatory genes such as *S100A12*, *S100A8*, *S100A9*, and *VCAN* was significantly increased in cells of state one. However, the expression of HLA family genes (*HLA-DRA*, *HLA-DRB1*, *HLA-DPA1*, *HLA-DPB1*) was low, suggesting a low antigen-presenting ability of these monocytes. The expression of *IFITM2* and *IFITM3* was decreased, indicating a weaker signal associated with type I interferon, which may be responsible for the diminished antigen-presenting capacity of monocytes. In addition, the expression of *FCGR3A*, *C1QA*, and *C1QB* was also downregulated, suggesting that monocytes in state one did not seem to be sensitive to adaptive immune responses. Subsequently, state one monocytes differentiated into those characterized by states two and three. In state two, the expression of the HLA family (*HLA-DRB5*, *HLA-DRA*, *HLA-DRB1*) genes was upregulated, while *FCGR3A*, *C1QA*, and *C1QB* showed low expression. Interestingly, the characteristics of state three were in contrast to those of state one. In state three, *FCGR3A*, *C1QA*, *C1QB*, *HLA-DPB1*, *HLA-DPA1*, *IFITM2*, and *IFITM3* were expressed at higher levels, while *S100A9*, *S100A8*, *S100A12*, and *LYZ* were all expressed at lower levels.

### Establishment of a prognostic signature related to monocyte differentiation in patients with sepsis

**Fig. 3:**
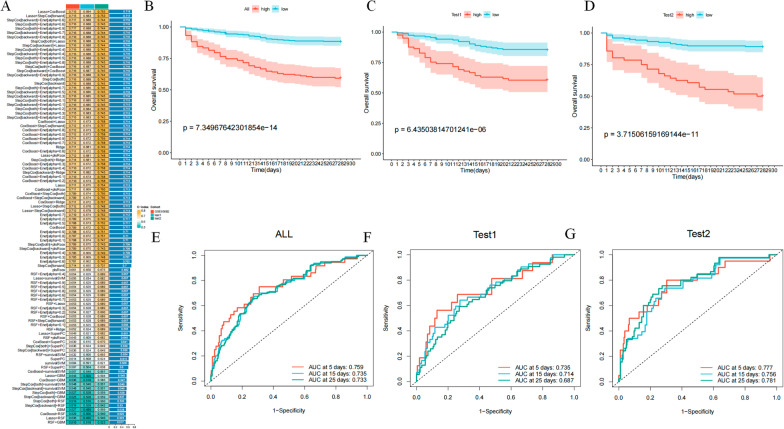
Establishment of a prognostic signature related to monocyte differentiation in patients with sepsis. **A** C-index values of 99 machine learning combinations. The color from green to yellow represents the gradual increase of C-index value. Each row represents a signature and each column represents a cohort; **B** Survival analysis of the total cohort of high-risk and low-risk patients. Blue represents low-risk patients and red represents high-risk patients; **C** Survival analysis of the Test 1 cohort of high-risk and low-risk patients. Blue represents low-risk patients and red represents high-risk patients; **D** Survival analysis of high-risk and low-risk patients in the Test 2 cohort; **E** ROC analysis of risk scores for the total cohort. Red represents the AUC of signature at 5 days, blue represents the AUC of signature at 15 days, and green represents the AUC of signature at 25 days; **F** ROC analysis of risk scores for the Test 1 total cohort. Red represents the AUC of signature at 5 days, blue represents the AUC of signature at 15 days, and green represents the AUC of signature at 25 days; **G** ROC analysis of the risk scores of the total cohort in Test 2. Red represents the AUC of signature at 5 days, blue represents the AUC of signature at 15 days, and green represents the AUC of signature at 25 days

Single-cell transcriptome sequencing data based on 12 patients with sepsis provide great assistance in the analysis of monocyte differentiation trajectories at the individual cell level. However, in contrast to bulk transcriptome sequencing, single-cell data are missing essential clinical information, such as patient survival information. To determine the prognostic value of 166 monocyte differentiation-related genes, we downloaded the GSE65682 dataset with survival information. The GSE65682 dataset comprises peripheral blood transcriptome sequencing data of 760 patients with sepsis and 42 healthy donors. We first used the transcriptome data of 469 patients with sepsis who had a clear 28-day record of survival status. We performed univariate Cox regression analysis on 166 monocyte differentiation-related genes and identified 51 prognosis-related genes. Among them, 42 genes were classified as protective genes and 9 genes as risk genes. These 51 prognosis-related genes were subjected to our machine learning integration process. Specifically, we used random survival forest (RSF), elastic network (Enet), Lasso, Ridge, stepwise Cox, CoxBoost, partial least squares regression for Cox (plsRcox), supervised principal components (SuperPC), generalized boosted regression (GBM), and survival support vector machine (survival-SVM). In the process, we used one algorithm to filter the variables and another to build the prognostic signature. When the number of genes included in the final prognostic signature was less than 5, the signature was considered invalid. Also, for signature accuracy validation, we randomly divided the 469 case samples into two validation cohorts, namely, test 1 and test 2, in a 1:1 ratio. Finally, we successfully fitted 99 signature combinations (Fig. 3A). For each signature, the Harrell’s concordance index (C-index) was calculated across all validation datasets, and the signature with the highest average C-index was considered optimal (Fig. 3A). Interestingly, among all signature combinations, the Lasso + CoxBoost signature with 17 genes showed the best prognostic prediction performance (Fig. 3A). Among all patient cohorts, the C-index value of Lasso + CoxBoost was 0.715. Furthermore, the C-index for test 1 and test 2 were 0.684 and 0.755, respectively.

### Signature validity and stability analysis

Based on the expression of these 17 genes, a risk score was calculated for each patient using the predict function. Risk score = (− 0.23041 × *LST1* expression) + (− 0.13571 × *LYZ* expression) + (0.19471 × *YBX1* expression) + (− 0.16417 × *MTSS1* expression) + (− 0.28547 × *SELL* expression) + (− 0.07437 × *ABI3* expression) + (0.22436 × *C1QA* expression) + (0.49227 × *RNASET2* expression) + (− 0.28756 × *NUP214* expression) + (− 0.13198 × *LILRA1* expression) + (0.23376 × *PPDPF* expression) + (0.25875 × *RHOB* expression) + (0.25079 × *CLEC12A* expression) + (0.15692 × *INSIG1* expression) + (− 0.18470 × *EVL* expression) + (0.39340 × *NDUFB1* expression) + (0.23379 × *BCL2A1* expression).

Subsequently, we further analyzed the validity of the risk scores in the total cohort, test 1 cohort, and test 2 cohort, respectively. First, patients in the total cohort, test 1 and test 2 cohorts were divided into high-risk and low-risk groups based on the risk score of each patient using the “srvminer” package to determine the optimal threshold value. In the total cohort and test 1 and test 2 cohorts, the 28-day overall survival (OS) was significantly lower in the high-risk patients (Fig. 3B–D), and the receiver operating characteristic (ROC) analysis showed that the area under curve (AUC) values for risk scores in the total cohort were 0.759, 0.735, and 0.733 at 5, 15, and 25 days, respectively. The AUC values for risk scores in test 1 and test 2 cohorts could still reach 0.735, 0.714, 0.687 and 0.777, 0.756, 0.781 at 5, 15, and 25 days (Fig. 3E, F). In summary, our results showed that the risk score could demonstrate stable and robust performance across multiple cohorts.

### Characterization of high- and low-risk patients

**Fig. 4:**
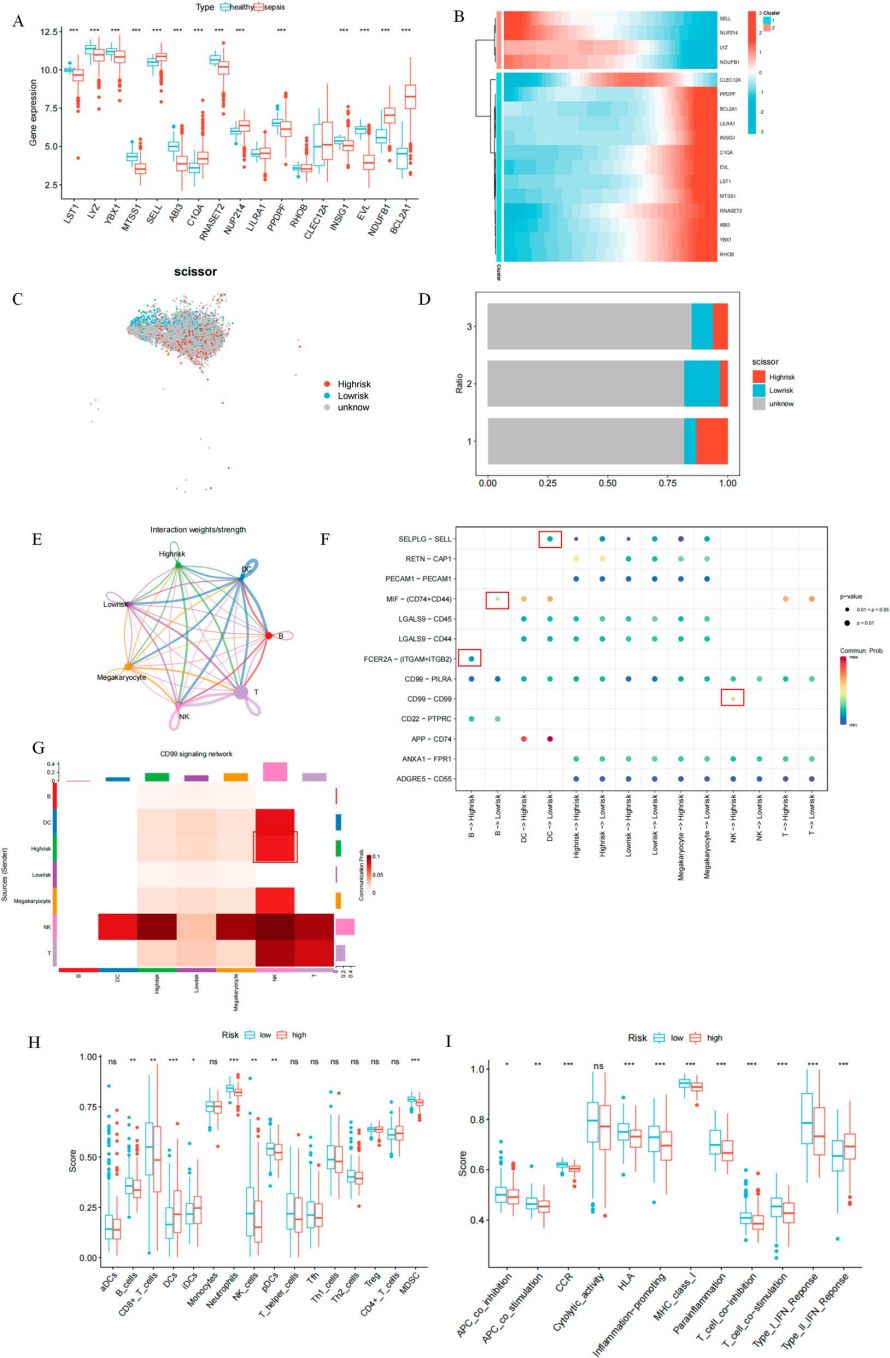
Characterization of high- and low-risk patients. **A** Differential expression of 17 signature gene between healthy individuals and sepsis patients. *P < 0.05, **P < 0.01, ***P < 0.001, ****P < 0.0001 by two-tailed t-test; **B** Expression of 17 signature gene in monocyte differentiation trajectory. Each row is a gene, each column is a sample, and the differentiation time is from left to right. The color changes from blue to red indicating increasing levels of gene expression; **C** Identification of cell subpopulations highly correlated with the phenotypes of high-risk and low-risk patients. Blue are identified low-risk cells, red are identified high-risk cells, and gray are unclassified cells; **D** Proportional analysis of phenotype-associated cell subpopulations in three differentiation states. The horizontal axis is the percentage of cells and the vertical axis is the different states of monocytes. Blue are identified low-risk cells, red are identified high-risk cells, and gray are unclassified cells; **E** Cellular communication landscape. Different colors represent that the signal originates from different cells, the thicker the line represents the higher weight of the signal action, and the arrows represent the direction of signal sending and receiving; **F** Analysis of ligand-receptor communication between different immune cells. The horizontal axis is the ligand cell and the corresponding receptor cell, and the vertical axis represents the different signal pairs. The color of the dots from blue to red represents the stronger the strength of the signal; **G** Cellular communication analysis of *CD99* signaling; **H** Analysis of immune cell content. Low-risk patients in blue and high-risk patients in red; **I** Analysis of immune function. Low-risk patients in blue and high-risk patients in red. *P < 0.05, **P < 0.01, ***P < 0.001, ****P < 0.0001 by two-tailed t-test

To investigate the internal characteristics of patients in the high-risk and low-risk groups, we first analyzed the differential expression of 17 genes involved in the signature construction between healthy and sepsis samples, of which 14 genes were differentially expressed (Fig. 4A). The expression of nine and five genes was down-regulated and up-regulated, respectively. We then analyzed the expression trends of the 17 genes during monocyte differentiation (Fig. 4B). We observed that *SELL*, *NUP214*, *LYZ*, and *NDUFB1* were highly expressed in the early stages of cell differentiation. These genes are associated with the promotion of leukocyte migration to secondary lymphoid organs and sites of inflammation, lysozyme secretion, and other effects (Bernimoulin et al. 2003; Mehta-D’souza et al. 2017). *CLEC12A* expression was upregulated in the intermediary stages of cell differentiation. This gene encodes a member of the C-type lectin/C-type lectin-like structural domain (CTL/CTLD) superfamily, which participates in cell adhesion, intercellular signaling, and inflammatory and immune responses (Marshall et al. 2006). The remaining genes, such as *EVL* and *BCL2A1* were highly expressed at the end of differentiation. These genes may be associated with the secretion of inflammatory cytokines, such as TNF and IL-1, at this stage. We aimed to identify monocytes that contribute to the high-risk phenotype for disease. Therefore, the “scissor” package was used to correlate bulk sequencing data with single-cell sequencing data. This method uses single-cell data and phenotypic information to identify subpopulations of cells. Using a large amount of data and its annotated information with various types of phenotypes, the algorithm automatically selects cells that are highly correlated with the phenotype. We considered high-risk and low-risk in patients as two phenotypes, associating both phenotypes with 7631 monocytes. We identified a total of 715 high-risk cells and 597 low-risk cells (Fig. 4C). We performed a reliability significance t-test using the “reliability.test” function in the “scissor” package. The results showed a correlation of t = 0.896 (P < 0.0001). This finding validates the accuracy of our cell identification. The results of the cell proportion analysis showed that the subpopulation of cells in status one had the highest percentage of high-risk cells (Fig. 4D). This is consistent with our previous results indicating that the cell subpopulation in state one showed a low expression of HLA family genes and a low antigen-presenting capacity. In addition, we analyzed the communication relationships between high- and low-risk cell subpopulations and other immune cells (Fig. 4E). We found significant differences between high-risk and low-risk cell subpopulations (Fig. 4F). For instance, FCER2A-(ITGAM + ITGB2) signaling was activated between B cells and high-risk cells. This signal activates a potent mitotic growth factor that plays an important role in the growth, differentiation, and regulation of IgE production in B cells. MIF-(CD74 + CD44) signaling was activated between B cells and low-risk cells. This finding suggests that the regulation of monocyte function in host defense, initiation of cell proliferation (Oddo et al. 2005). SELPLG-SELL signaling were activated between DC cells and low-risk cells. This signal plays a key role in the transport of leukocytes and the subsequent binding (Sako et al. 1995). CD99–CD99 signaling was activated between NK cells and high-risk cells (Fig. 4G). CD99-encoded protein can be involved in mediating cell death via a caspase-independent pathway (Hahn et al. 1950). Finally, we analyzed the immune microenvironment of patients in the high and low-risk groups. We calculated the immune cell function and immune cell content of patients in the high and low-risk groups using the ssGSEA method. The results of the immune cell content showed a decrease in the levels of several T cells in the high-risk group, such as CD8T cells and T helper cells. In addition, the levels of NK cells and B cells were significantly downregulated (Fig. 4H). These findings suggest that high-risk patients may be in a state of immune cell depletion. Immune function analysis showed that antigen-presenting ability, pro-inflammatory ability, T cell co-activation and co-inhibitory ability were all downregulated in high-risk patients (Fig. 4I). Interestingly, IFN-I responses were down-regulated while IFN-II responses were up-regulated, which also remained consistent with state one cells.

### EVL showed a stable diagnostic and prognostic value

**Fig. 5:**
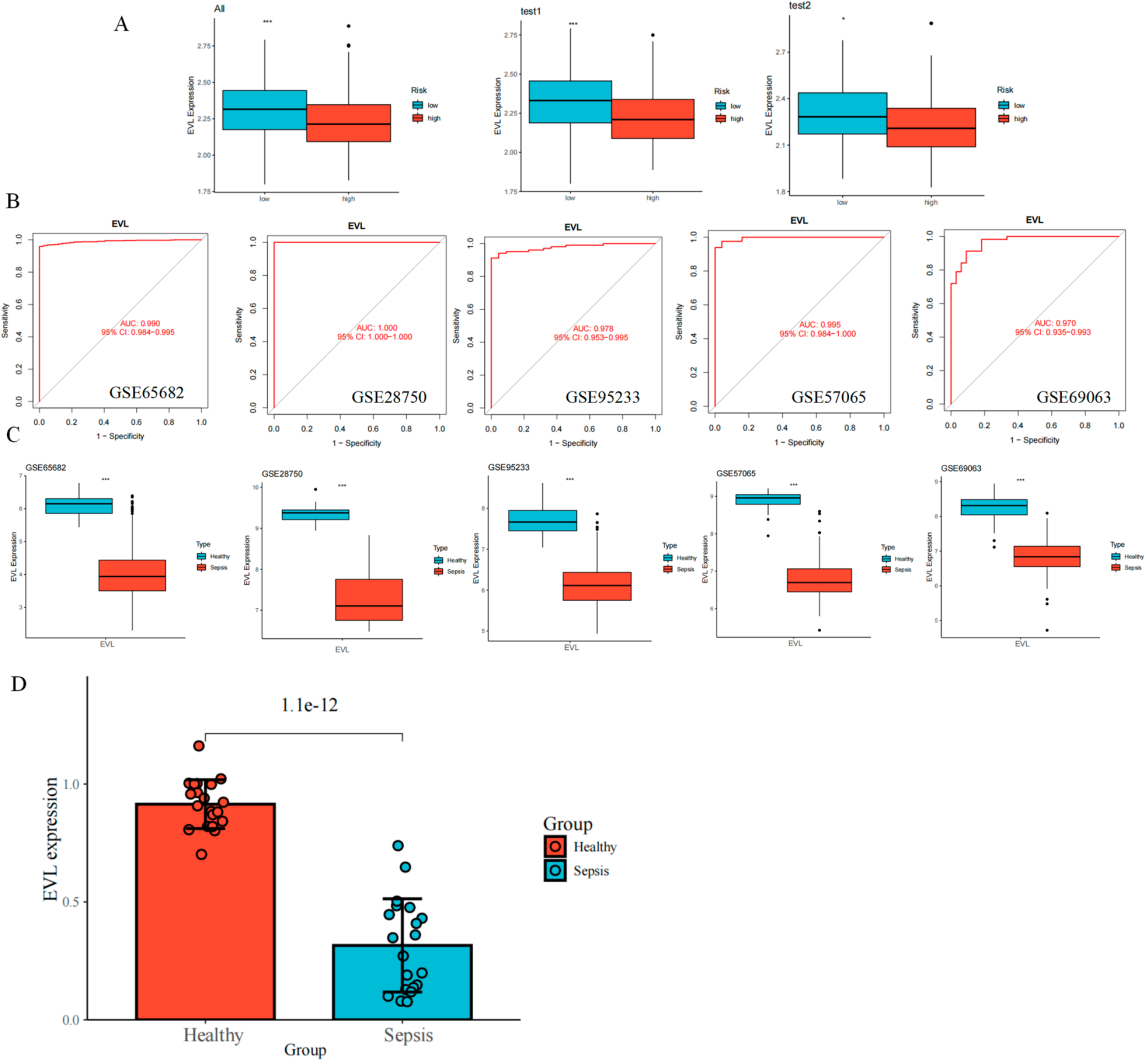
Analysis of the diagnostic and prognostic value of *EVL*. **A** Expression of *EVL* in high-risk and low-risk patients. Low-risk patients in blue and high-risk patients in red; **B** ROC curves of EVL in different datasets; **C** Differential expression of *EVL* among healthy patients with any sepsis. Blue is for healthy people and red is for sepsis patients; **D** Clinical sample validation of *EVL* differential expression in healthy individuals and sepsis patients. Red for healthy people and blue for septic patients. *P < 0.05, **P < 0.01, ***P < 0.001, ****P < 0.0001 by two-tailed t-test

We aimed to identify a gene with both diagnostic and prognostic value, which may be more important for clinical purposes. Among the genes involved in the construction of our prognostic signature, the *EVL* gene in the signature had protective functions. In addition, we noted that *EVL* expression was low in high-risk patients in all cohorts, test 1 and test 2 cohorts (Fig. 5A). Importantly, as a diagnostic marker, *EVL* could reach an AUC value of 0.990 in the GSE65682 dataset (Fig. 5B). To further determine the stability of the diagnostic value of *EVL*, we analyzed GSE28750, GSE95233, GSE57065, and GSE69063. The baseline characteristics of the samples in these datasets are shown in Additional file 1: Table S1. GSE28750 included 20 healthy samples and 10 sepsis samples. GSE95233 included 22 healthy samples and 102 sepsis samples. GSE57065 included 25 healthy samples and 82 sepsis samples. GSE69063 included 33 healthy samples and 57 sepsis samples. Our results showed that the AUC of *EVL* was 1 in GSE28750, 0.978 in GSE95233, 0.995 in GSE57065, and 0.970 in GSE69063 (Fig. 5B), and *EVL* expression was downregulated in patients with sepsis compared to that in healthy samples in all datasets (Fig. 5C). To validate the altered *EVL* expression in patients with sepsis in the real world, we enrolled a clinical cohort of 20 healthy individuals (mean age, 56 years; 66% men) and 20 patients with sepsis (mean age, 58 years; 66% men). *EVL* expression were significantly lower in the cohort of sepsis patients than in healthy donors (Fig. 5D). In conclusion, *EVL* demonstrated a stable diagnostic value for sepsis and that the downregulation of *EVL* may be related to disease progression in patients with sepsis. However, the causal relationship between *EVL* and sepsis progression remains largely unknown.

### EVL-related gene regulatory networks

**Fig. 6:**
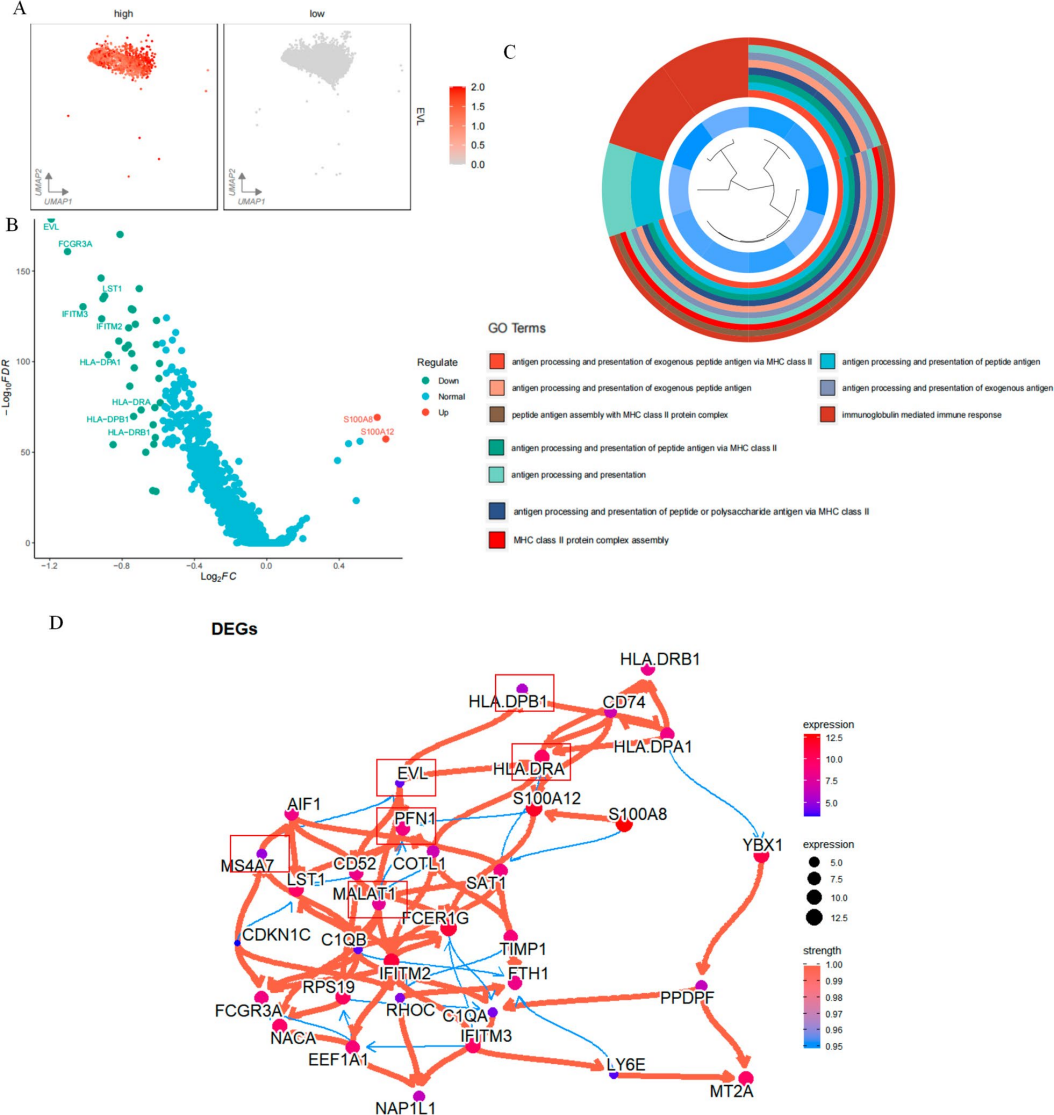
Analysis of the gene regulatory network of *EVL*. **A** *EVL* was divided into high and low expression groups by the presence or absence of *EVL* expression. The color changes from gray to red representing increasing gene expression; **B** Differential analysis of *EVL* low-expressing cells and EVL high-expressing cells. Green represents down-regulated genes, red represents up-regulated genes, and blue represents genes that did not change; **C** GO enrichment analysis. In the outermost circle, different colors represent different pathways. In the inner circle, each bar represents a gene, and the bluer the color, the lower the expression value of that gene; **D** Bayesian network to infer the gene regulatory network of *EVL.* Each node represents a gene, the redder the node color, the larger the node means the higher the expression value of the gene. The thicker the line is, the greater the strength of the regulatory effect

We divided all monocytes into *EVL* low-expression group and *EVL* high-expression group based on whether they expressed *EVL* or not (Fig. 6A). We performed differential analysis between the low and high expression groups and identified 39 differential genes in the low expression group (|log2FC| > 0.585, adjusted P < 0.05) (Fig. 6B). Interestingly, *FCGR3A*, *IFITM3*, *HLA-DAPA*, *HAL-DPB1*, and *HLA-DRA* were down-regulated, and *S100A8* and *S100A12* were up-regulated. This finding was consistent with the characteristics of state one cells. Once again, we demonstrated that cells in state one may be key for identifying poor prognosis in patients with sepsis. We subjected these differential genes to Gene ontology (GO) enrichment analysis, which showed that a large number of downregulated genes were enriched in antigen-presentation-related pathways (Fig. 6C). This finding remains consistent with our hypothesis that low expression of *EVL* promotes impairment of monocyte antigen-presentation capacity. To explore unelucidated roles in this regulatory network as accurately as possible, we used a “CBNplot” package for the inference of interactions between genes. The software uses a computational approach based on Bayesian networks to infer directed regulatory relationships between genes. The method is efficient, as analyzed based on currently known regulatory roles and published literature. In our putative regulatory network (Fig. 6D), *EVL* directly regulates *HLA-DPB1*, *HLA-DRA*, *MALAT1*, and *PFN1*. *MS4A7* acts as an upstream gene to regulate the expression of EVL. In conclusion, the results of these analyses suggest a mechanism by which the downregulation of *EVL* leads to the downregulation of HLA family genes, which leads to low monocyte antigen presentation and ultimately affects the prognostic status of patients with sepsis.

## Discussion

Septicemia, which refers to “decay” in Greek, is considered a common wound complication (Evans 2018). The definition of sepsis is still very difficult to elucidate (Simonsen et al. 2014). Although intensive care medicine and antibacterial treatment have made significant progress, sepsis is still a common disease with high mortality (Dellinger et al. 2013; Minasyan 2019). Patients most affected by sepsis need to be treated in the intensive care unit (Evans 2018; Hunt 2019). The condition of patients with sepsis changes rapidly, and a delay of each hour in administering appropriate antibiotic treatment can lead to a 4–7% increase in case fatality. Therefore, early diagnosis and intervention may help improve the prognosis of patients with sepsis (Arina and Singer 2021; Evans 2018; Emr et al. 2018). The early diagnosis and treatment of patients with sepsis is the main problem (Suetrong and Walley 2016; Jain 2018). To find better biomarkers for the early detection and treatment of sepsis, we collected sequencing data of peripheral blood mononuclear cells derived from patients with sepsis. The differentiation sites of monocytes were analyzed, and the differentiation-related genes were identified. Based on the expression of differentiation-related genes, a combination of prognostic features based on machine learning methods was constructed, and the best machine learning algorithm was used to calculate the risk score of all patients. The validity and accuracy of the best model were systematically analyzed. Then, the diagnostic value and prognostic value of EVL were determined. The results of 40 samples were clinically verified. The data provide a reference for clinical diagnosis and treatment.

First, we obtained the sequencing data of peripheral blood single cells derived from 12 patients with sepsis and annotated all cells. Monocytes accounted for more than 50% of all cells. Three distinct differentiation states of monocytes were identified. The low expression of HLA family genes (*HLA-DRA*,* HLA-DRB1*,* HLA-DPA1*,* HLADPPB1*) in state one cells suggests that these monocytes had a low antigen-presenting ability and do not seem to be sensitive to adaptive immune responses. Subsequently, monocytes in state one differentiated into those in state two and state three. The HLA family (*HLA-DRB5*,* HLA-DRA*,* HLA-DRB1*) genes were up-regulated in state two cells. Interestingly, the characteristics of state three cells were in contrast to those of state one cells. In state three, *FCGR3A*,* C1QA*,* C1QB*,* HLA-DPB1*,* HLA-DPA1*,* IFITM2*, and *IFITM3* were highly expressed, while the expression of *S100A9*,* S100A8*,* S100A12*, and* LYZ* was low. This is similar to the previous report demonstrating different states of immune cells during sepsis (Ming et al. 2019). Some cells are pro-inflammatory, while others inhibit the onset of inflammation. These immune cells are often the primary factors responsible for the development of sepsis (Rimmelé et al. 2016; Boomer et al. 2011). Monitoring the expression of *HLA-DR* can aid in the diagnosis, prognosis, and prediction of sepsis (Zhuang et al. 2017; Kyriazopoulou and Giamarellos-Bourboulis 2021; Yoshida 2004; Pandey et al. 2021; Xu et al. 2020; Boeddha et al. 2018; Lelubre et al. 2017). Two studies published in 2022 also examined the relationship and mechanism associated with the expression of HLA family proteins and sepsis (Horn et al. 2022; Wu et al. 2022). In general, during sepsis, monocytes in the body of patients differentiate into those with different states along with progress in the condition of the patient, which also plays an important role in the development of sepsis (Boeddha et al. 2018; Liu et al. 2021; Silva et al. 2013). In particular, the ability of antigen presentation is of great significance for the diagnosis, prognosis, and prediction of sepsis in patients (Silva et al. 2013; Quadrini et al. 2021; Hahn et al. 2017; Fernández-Grande et al. 2019).

Subsequently, we established a prognosis signature related to monocyte differentiation in patients with sepsis. Transcriptome data of patients with sepsis with a defined 28-day survival status were used. A total of 51 prognosis-related genes were identified. We accepted the integration process of machine learning and selected the most effective machine learning algorithm to build a prognosis signature. The results of cell proportion analysis showed that the high-risk cells accounted for most cells in the subpopulation of state one cells. This is consistent with our previous results; expression of the HLA family genes in state one cell subsets was low, along with reduced antigen presentation ability. In addition, the results of cell communication were also consistent with those reported in previous articles. The FCER2A − (ITGAM + ITGB2) signal between B cells and high-risk cells was activated. The MIF −  (CD74 + CD44) signal between B cells and low-risk cells was activated (Bu et al. 2020; Parvaneh et al. 2010; Wen et al. 2022; Tilstam et al. 2021; Martin 2000). The SELPLG-SELL signal between DC cells and low-risk cells was activated (Bime et al. 2018). The CD99-CD99 signal between NK cells and high-risk cells was activated (Cruz et al. 2004). Finally, for the immune microenvironment, we calculated the immune cell function and immune cell content of patients in high-risk groups. Immune cell analysis showed that the number of various T cells, such as CD8T cells and T helper cells, in the high-risk group decreased. In addition, NK cell levels were also significantly reduced. The results suggest that high-risk patients may be in a state of immune cell depletion (Cruzat et al. 2018; He et al. 2021; Nakamori et al. 2020).

Finally, we identified a gene *EVL* with both diagnostic and prognostic value. To determine the diagnostic value of *EVL*, the gene expression was verified by GEO data and clinical samples. *EVL* showed a stable diagnostic value for sepsis, and the down-regulation of EVL may be related to the disease progression in patients with sepsis. However, the causal relationship between *EVL* and sepsis progression remains largely unknown. We divided monocytes into high and low *EVL* expression groups. A total of 39 differential genes were identified (|log2FC| > 0.585, P < 0.05 after adjustment). Interestingly, the expression of *FCGR3A*,* IFITM3*,* HLA-DAPA*,* HAL-DPB1*, and* HLA-DRA* was down-regulated and that of *S100A8* and* S100A12* was up-regulated. This was consistent with the characteristics of state one cells. State 1 may be the key cell state with poor prognosis in patients with sepsis. The enrichment analysis showed that a large number of downregulated genes were enriched in the antigen production-related pathway. This was consistent with our hypothesis stating that the low expression of *EVL* promoted the damage in monocyte antigen presentation ability. Therefore, in the regulatory network, we speculated that *EVL* directly regulated *HLA-DPB1*, *HLA-DRA*, *MALAT1*, and *PFN1.* As an upstream gene, *MS4A7* regulates the expression of *EVL*. In conclusion, these results suggest that the downregulation of *EVL* leads to the downregulation of the expression of HLA family genes, which, in turn, results in reduced monocyte function and ultimately affects the prognosis of patients with sepsis.

It is important to note that the prevention of sepsis is multifaceted. Early identification and prompt treatment of infections with appropriate antibiotics or other therapies can effectively control infections before they develop into sepsis in many cases. Proper antibiotic use is another important strategy to prevent sepsis, but it must be used judiciously to avoid the emergence of antibiotic-resistant bacteria (Rhee et al. 2020). We recommend that healthcare providers prescribe antibiotics only when truly necessary, and choose the most appropriate antibiotics for specific infections. Vaccination is also an important strategy for preventing sepsis. Vaccines can reduce the risk of infection from certain diseases (such as influenza, pneumonia, and meningococcal disease) that can lead to sepsis, thereby reducing the risk of sepsis (Richardson et al. 2014). Infection control measures are also key to preventing sepsis. These measures include practices such as hand hygiene, the use of personal protective equipment, and isolation precautions for infectious patients. These measures can help prevent the spread of infections in healthcare settings and communities. In addition to these strategies, exploring the use of new antibiotics, immunotherapies, or other treatments to prevent infections from progressing to sepsis may be necessary in the future. Improving surveillance and monitoring systems is also an important step in preventing sepsis, which involves developing better tools and methods for detecting and tracking cases of infection and sepsis. By improving surveillance and monitoring, healthcare providers can detect cases of sepsis earlier and provide more timely and effective treatment. Finally, raising awareness of sepsis and its prevention is crucial for healthcare institutions and the public. Education and awareness campaigns can help ensure that healthcare providers are aware of sepsis and take appropriate measures to prevent it. At the same time, public awareness campaigns can help people understand the importance of preventive measures for infection, as well as early recognition and treatment of infections.

In conclusion, to perform early detection, diagnosis, and treatment of sepsis, we examined associated biomarkers. We not only found the best prognosis model through a variety of machine learning algorithms but also finally determined the gene *EVL* with diagnostic value and prognostic value. The results were verified at clinical levels in patients. The findings provide a certain reference for clinical diagnosis and treatment and a direction for the exploration for related mechanisms.

## Conclusions

Monocyte differentiation-related prognostic signatures based on the Lasso + CoxBoost combination were able to accurately predict the prognostic status of patients with sepsis. In addition, low *EVL* expression was associated with poor prognosis in sepsis.

## Supplementary Information

**Additional file 1: Table S1.** The baseline information of the samples used in the study.

## Data Availability

Single-cell transcriptome data were obtained from the Single Cell Portal (https://singlecell.broadinstitute.org/single_cell/study/SCP548/an-immune-cell-signature-of-bacterial-sepsis-patient-pbmcs#study-summary). Bulk transcriptome sequencing data were obtained from the GEO database, GEO registration numbers are GSE65682, GSE69063, GSE28750, GSE95233, GSE57065.

## Abbreviations

SIRS: Systemic inflammatory response syndrome
BBB: Blood–brain barrier
PAMPs: Pathogen-associated molecular patterns
PRR: Pattern recognition receptors
PCA: Principal component analysis
HSP: Examples include heat shock protein
HMGB-1: High mobility group box 1
EVL: Enah/Vasp-like
PCs: Principal components
GEO: Gene expression omnibus
GO: Gene Ontology
ROC: Receiver operating characteristic
AUC: Area under the curve
RSF: Random survival forest
Enet: Elastic network
plsRcox: Partial least squares regression for Cox
SuperPC: Supervised principal components
GBM: Generalised boosted regression
survival-SVM: Survival support vector machine
C-index: Concordance index
OS: Overall survival
